# Room-temperature field effect transistors with metallic ultrathin TiN-based channel prepared by atomic layer delta doping and deposition

**DOI:** 10.1038/s41598-017-00986-z

**Published:** 2017-04-13

**Authors:** Po-Hsien Cheng, Chun-Yuan Wang, Teng-Jan Chang, Tsung-Han Shen, Yu-Syuan Cai, Miin-Jang Chen

**Affiliations:** Department of Materials Science and Engineering, National Taiwan University, Taipei, Taiwan

## Abstract

Metallic channel transistors have been proposed as the candidate for sub-10 nm technology node. However, the conductivity modulation in metallic channels can only be observed at low temperatures usually below 100 K. In this study, room-temperature field effect and modulation of the channel resistance was achieved in the metallic channel transistors, in which the oxygen-doped TiN ultrathin-body channels were prepared by the atomic layer delta doping and deposition (AL3D) with precise control of the channel thickness and electron concentration. The decrease of channel thickness leads to the reduction in electron concentration and the blue shift of absorption spectrum, which can be explained by the onset of quantum confinement effect. The increase of oxygen incorporation results in the increase of interband gap energy, also giving rise to the decrease in electron concentration and the blue shift of absorption spectrum. Because of the significant decrease in electron concentration, the screening effect was greatly suppressed in the metallic channel. Therefore, the channel modulation by the gate electric field was achieved at room temperature due to the quantum confinement and suppressed screening effect with the thickness down to 4.8 nm and the oxygen content up to 35% in the oxygen-doped TiN ultrathin-body channel.

## Introduction

With the rapid evolution of semiconductor technology down to the sub-10 nm technology node, nanoscale transistors based on new structures and materials have been explored^1–11^. One of the possible candidates for sub-10 nm devices is the junctionless transistors (JLTs), in which an ultrathin semiconductor body with a single doping type in the source, channel, and drain has been proposed^2, 12–15^. A JLT can be regarded as a “gated-resistor” with the absence of PN junctions in the source and drain regions. A gate voltage is applied to control the current by the formation of a depletion region in channel. Because of the absence of PN junctions in JLTs, the ultrafast thermal annealing for the dopant activation in source and drain is not needed, which allows one to fabricate the transistors with smaller channel lengths. Moreover, the presence of PN junctions in metal-oxide-semiconductor field effect transistors (MOSFETs) causes the extension of source/drain depletion charges into the channel, giving rise to severe short-channel effects including the drain-induced barrier lowering and degraded subthreshold slope^2^. Hence the severe short-channel effects can be alleviated in JLTs^15–17^. In addition, in the OFF state of JLTs, the effective distance between source and drain is longer than the physical gate length due to the extension of the channel depletion into source and drain, which suppresses the short channel effects in JLTs^17, 18^. Accordingly, the junctionless structure provides the solution to overcome the difficulties related to aggressive scaling of MOSFETs. Actually, the JLT with a channel length down to 3 nm has been demonstrated^19^. In order to achieve a high ON current of JLTs, a high doping concentration (>10^18^ cm^−3^) is required in the source, channel, and drain, which leads to the almost metallic nature of the channel in JLTs^2^. Because of the high doping concentration, the channel in JLTs has to keep sufficient thin for full depletion of carriers^15, 20^. However, for the JLTs with sub-10 nm channel lengths, the channel only comprises a few hundred or thousand atoms, and the incorporation of a few donors or acceptors results in a very high doping concentration. Thus the issue of threshold voltage variability induced by random dopants fluctuations arises^20–22^. Therefore, it is highly desirable to develop novel JLTs which are free of the random dopants fluctuations.

Metallic channels may be the promising solution for overcoming the random dopants fluctuations in JLTs. Unlike the semiconductor-based JLTs, the source, channel, and drain in metallic channel transistors are composed of metallic ultrathin bodies. The high electron concentration in metallic channels allows the channel free of the heavy doping as required in JLTs, and so the issue of random dopants fluctuations can be avoided. Besides, metallic channels also benefit from no Schottky barrier at the source and drain. In fact, the graphene, carbon nanotube, crystalline tin, and bismuth-based compounds have been proposed as the metallic channels, and the conductance modulation of such metallic channels by the electric field has been observed typically at low temperatures^23–29^. The key point of metallic channels is their high electron concentration, which prevents the penetration of electric field into the channel. Hence the screening effect leads to the difficulty in modulating the electron concentration by the electric field, giving rise to the poor gate control as the dimension of metallic channels is greater than the screening length. As a result, the thickness of metallic channels should be controlled carefully to be less than the screening length in order to achieve the field effect. In addition, the quantum confinement effect will take place if the metallic channel is thin enough. The split of energy levels caused by quantum confinement may result in the increase of the interband gap energy, which is beneficial to depletion of carriers in the channel to achieve the OFF state of transistors^30–33^.

Titanium nitride (TiN) is widely known as a metallic material with low electrical resistivity, great chemical stability, and high thermal conductivity^34^. Thin TiN films have been used as the metal gate and diffusion barrier in modern integrated circuits^35, 36^. In bulk TiN, there is no bandgap because of the overlapping between the conduction band and valence band. It has been observed that the electrical properties of metallic TiN thin films can be modified by the oxygen incorporation, which leads to the decrease of the electron concentration^37–40^. In addition, the bandgap can be opened in the oxygen-doped TiN if the oxygen content is more than 30%, as revealed by the first-principles density-functional calculations^34, 41^.

Atomic layer deposition (ALD) might play an important role in the ultrathin body of metallic channels. Because of the self-limiting growth of ALD, the ALD technique provides the following benefits: (1) Film thickness can be controlled precisely and digitally by the number of ALD cycles, (2) excellent uniformity, step coverage, and conformality, (3) low defect density without pinhole structures, (5) large-area and batch-type capability, and (6) high reproducibility due to wide processing windows because ALD is not sensitive to the variations and inhomogeneity of the temperature or precursor doses due to the self-limiting deposition. Therefore, the ALD technique is very suitable to precisely prepare the ultrathin body of metallic channel transistors. In addition, owing to the layer-by-layer (or “digital”) growth, the ALD technique has the *in-situ* capability of precise atomic layer incorporation of dopants to achieve high doping concentration and maintain atomic level control of the doping process. The atomic engineering of dopant incorporation might induce the modification of the band structure to get new electrical properties of nanoscale thin films.

In this study, the atomic layer delta doping and deposition (AL3D) technique was proposed to prepare the metallic channel transistors with the oxygen-doped TiN ultrathin-body channel. The metallic ultrathin-body channel must be thin enough and its oxygen content has to be sufficient high for the decrease of the electron concentration and the increase of the interband gap energy, which can be accurately controlled at the atomic scale by the layer-by-layer growth of AL3D. Besides, as a result of the self-limiting process of AL3D, the oxygen-doped TiN ultrathin-body channel might be free of the problems caused by the random doping fluctuations in semiconductor-based JLTs. With the decrease of film thickness and the increase of oxygen content, the reduction of the electron concentration and the blue-shift of optical absorption were observed, which indicates the suppression of screening effect and the increase of the interband gap energy. The significant modulation of channel resistance by the gate electric field were achieved at room temperature in the oxygen-doped TiN ultrathin-body channel.

## Materials and Methods

**Figure 1:**
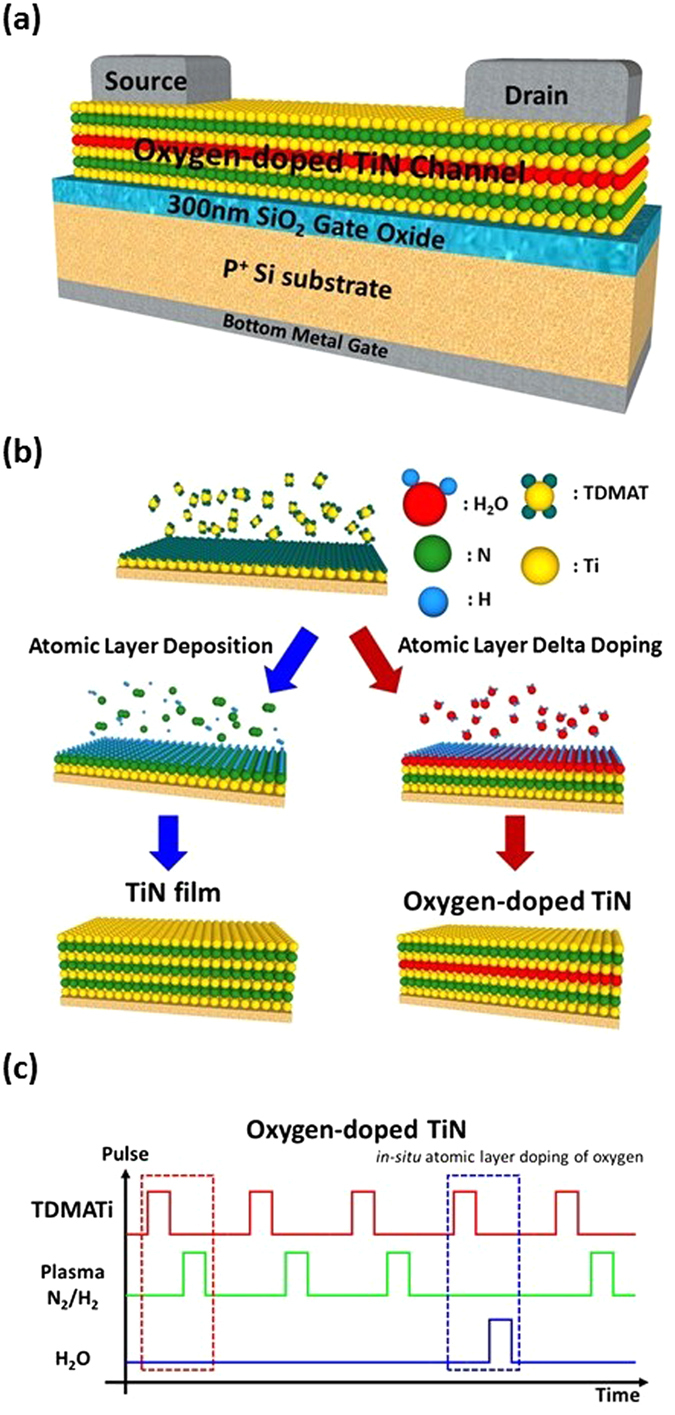
(**a**) The sketch of the bottom-gated metallic channel transistors with the oxygen-doped TiN ultrathin-body channel. (**b**) The schematic drawing of the AL3D process for preparing the oxygen-doped TiN ultrathin-body channel. (**c**) Illustration of the precursor pulse as a function of time in the AL3D process.

Figure 1(a) shows the schematic structure of the bottom-gated metallic channel transistors with the oxygen-doped TiN ultrathin-body channel. The substrate in this work was p^+^ Si with a heavy doping concentration of 5 × 10^19^ cm^−3^. A 300 nm SiO_2_ layer on the Si substrate was prepared by the thermal oxidation at 950^◦^C. Then an oxygen-doped TiN ultrathin body was deposited on the SiO_2_ layer at a temperature of 300^◦^C by the plasma-enhanced ALD (Ultratech, Fiji). The tetrakis(dimethylamino)titanium (TDMATi, Ti[N(CH_3_)_2_]_4_), N_2_/H_2_ plasma, and H2O vapor were used as the precursors for titanium, nitrogen, and oxygen, respectively (Fig. 1(b) and (c)). The AL3D process comprised two kinds of ALD cycles: (1) TDMATi → Ar purge → N_2_/H_2_ plasma → Ar purge for the deposition of one monolayer of TiN, and (2) TDMATi → Ar purge → H_2_O → Ar purge for the *in-situ* atomic layer delta doping of oxygen. Multiple ALD cycles for the *in-situ* atomic layer delta doping of oxygen were uniformly distributed in the total ALD cycles. The thickness and composition of the oxygen-doped TiN ultrathin body was controlled digitally by the number of applied ALD cycles in the AL3D process. For instance, as shown schematically in Fig. 1(c), if one ALD cycle for the *in-situ* atomic layer delta doping of oxygen was performed every 4 TiN ALD cycles, the oxygen-doped TiN layer with the nominal oxygen doping percentage (*DP*_*O*_) of 20% was obtained. Afterwards, the thermal evaporation and lift-off process were used to form the Cr/Au contacts at the source and drain. Finally, the source, channel, and drain of the transistor with the channel width and length of ~1 and 200 µm, respectively, were defined by the optical lithography and wet etching.

The Hall effect measurement (Ecopia HMS-3000) was used to probe the resistivity and electron concentration of the oxygen-doped TiN ultrathin body. The thickness and extinction coefficients were obtained by the spectroscopic ellipsometer (SE, Elli-SE, Ellipso Technology), according to which the absorption spectra of the oxygen-doped TiN ultrathin body were extracted. The chemical compositions of the oxygen-doped TiN layers were measured by the Auger electron spectroscopy (AES, JAMP-9510F Field Emission Auger Microprobe, JEOL). The Keysight B1500A semiconductor device analyzer was utilized to measure the current-voltage characteristics of the metallic channel transistors at room temperature.

## Results and Discussion

**Figure 2:**
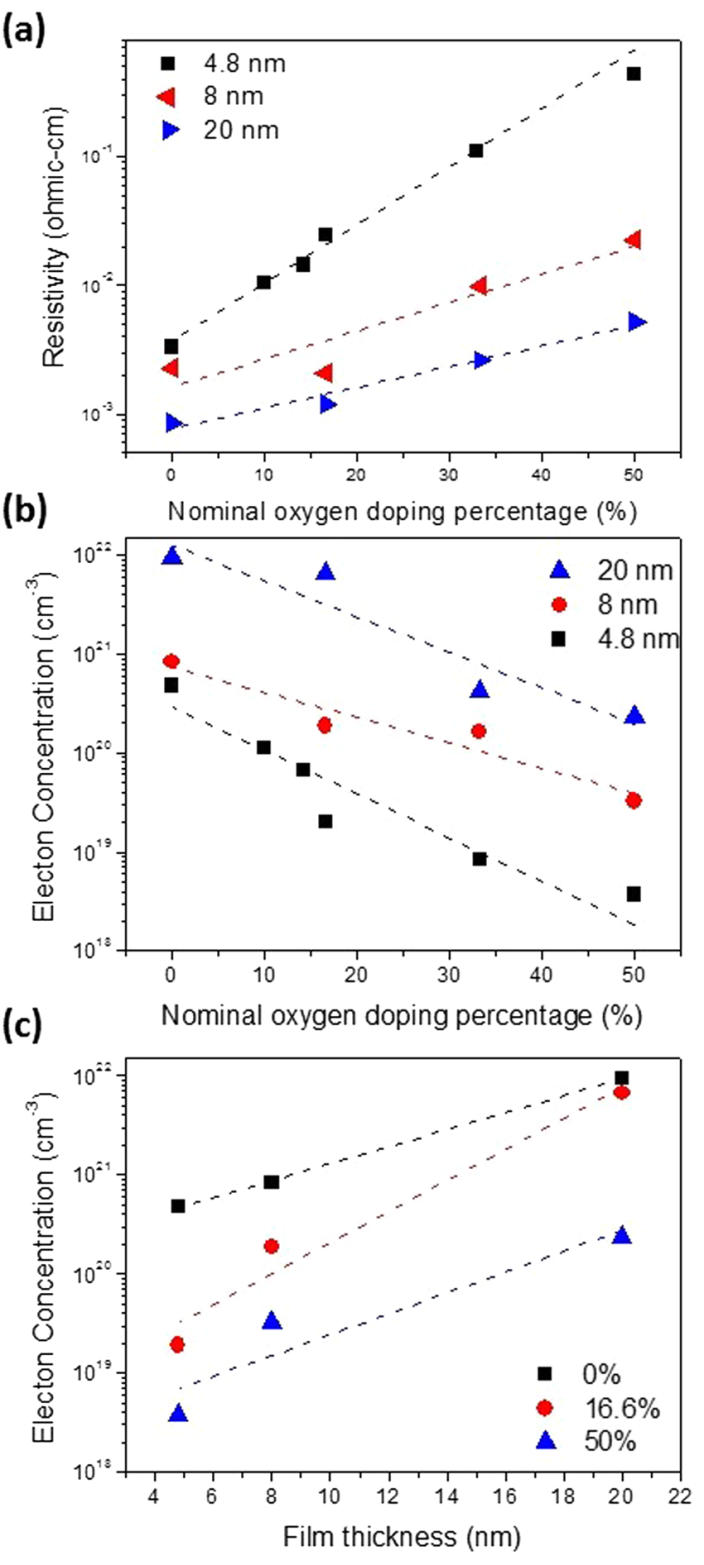
Electrical characteristics of the oxygen-doped TiN layers. (**a**) The resistivity as a function of the nominal oxygen doping percentage (*DP*_*O*_) of the layers with different thicknesses. (**b**) The electron concentration as a function of the *DP*_*O*_ in the layers with different thicknesses. (**c**) The electron concentration as a function of the film thickness in the layers with different *DP*_*O*_.

The resistivity and electron concentration of the oxygen-doped TiN layers with different thickness as a function of the nominal oxygen doping percentage (*DP*_*O*_) are presented in Fig. 2(a) and (b). The 20 nm TiN layer without the *in-situ* atomic layer delta doping of oxygen (*DP*_*O*_ = 0%) exhibited a low resistivity and a high electron concentration of 8.6 × 10^–4^ Ohmic-cm and 9.4 × 10^21^ cm^−3^, respectively. The increase of *DP*_*O*_ results in the increment of the resistivity and the reduction in the electron concentration of the oxygen-doped TiN layers. The decrease of the electron concentration could contribute to the suppression of screening effect in the oxygen-doped TiN layers. Figure 2(c) shows the thickness dependence of the electron concentration in the oxygen-doped TiN layers with different *DP*_*O*_, indicating that the electron concentration in all the films decreases with the reduction in the film thickness. Actually, the blue shift of the PL spectra of the TiN nanoparticles has been observed with the decreasing particle sizes from 20 nm to 5.4 nm, which was deduced from the quantum confinement effect^42–47^. In addition, it has been reported that the quantum confinement effect would induce the metal-semimetal transition as the TiN thickness is scaled down to the critical dimension^48^. Hence, the decrease in the electron concentration in the oxygen-doped TiN layers might be explained by the reduction in the density of states with the decreasing film thickness because of the quantum confinement effect.

**Figure 3:**
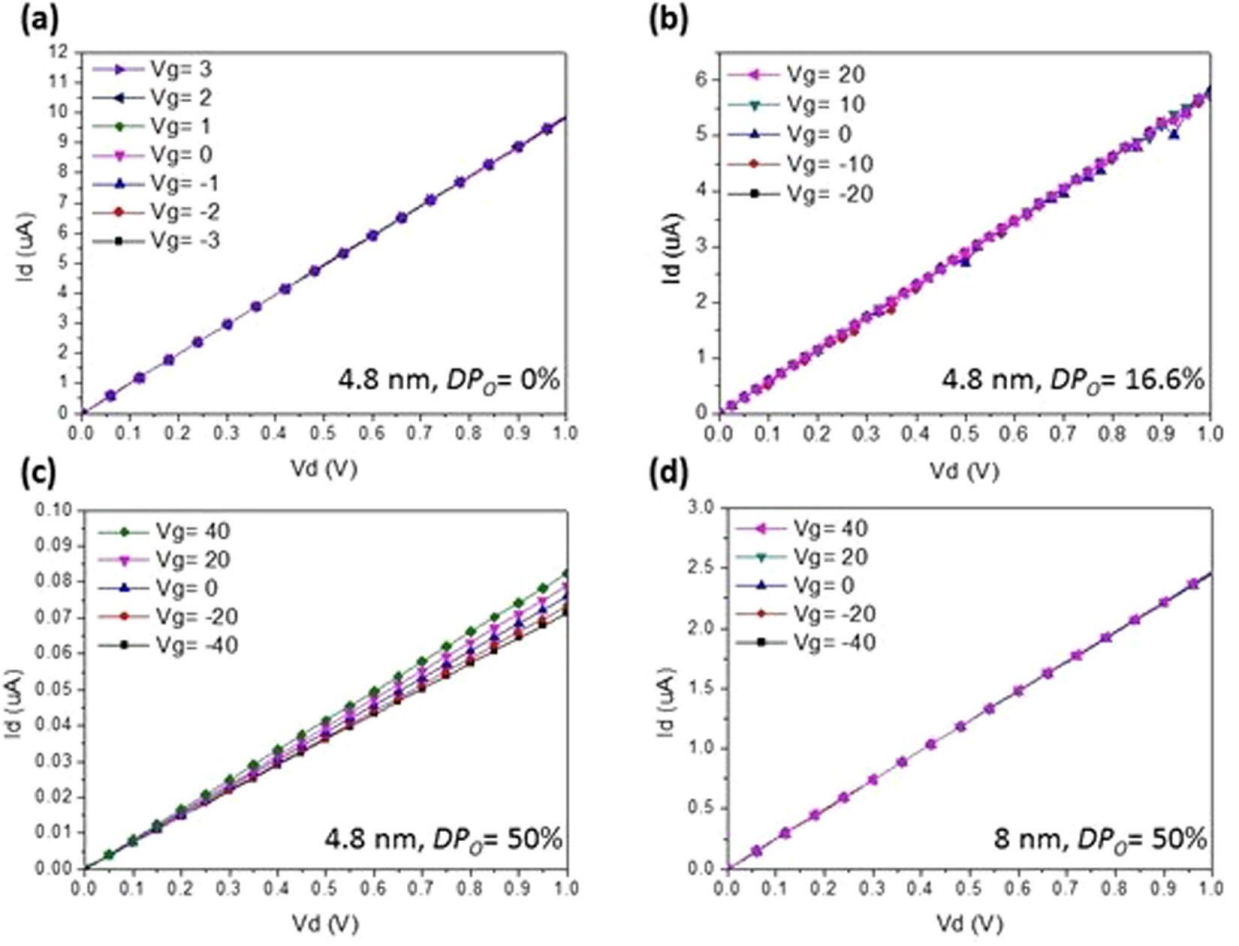
Room-temperature *I*_*d*_-*V*_*d*_ characteristics at various bottom gate voltages (*V*_*g*_) of metallic channel transistors with the oxygen-doped TiN ultrathin-body channel. (**a**) 4.8 nm TiN-based channel with the nominal oxygen doping percentage (*DP*_*O*_) of 0%. (**b**) 4.8 nm TiN-based channel with the *DP*_*O*_ of 16.6%. (**c**) 4.8 nm TiN-based channel with the *DP*_*O*_ of 50%. (**d**) 8 nm TiN-based channel with the *DP*_*O*_ of 50%.

Figure 3 shows the characteristics of drain current (*I*_*d*_) versus drain voltage (*V*_*d*_) at various bottom gate voltages of metallic channel transistors with the oxygen-doped TiN ultrathin-body channel at room temperature. In Fig. 3(a) and (b), when the bottom gate voltage was applied on the 4.8 nm TiN-based channel with the nominal oxygen doping percentage (*DP*_*O*_) of 0% and 16.6%, the *I*_*d*_ were not modulated at all by the gate electric field. The result indicates the metallic nature of the 4.8 nm TiN-based channel with the *DP*_*O*_ of 0% and 16.6%, which is consistent with their high electron concentrations (4.8 × 10^20^ and 2 × 10^19^ cm^−3^, respectively) as revealed in Fig. 2 (b). As the *DP*_*O*_ increases to 50%, the electron concentration decreases to 3.7 × 10^18^ cm^−3^ in the 4.8 nm oxygen-doped TiN ultrathin-body channel, which results in the significant *I*_*d*_ modulation by the bottom gate voltage as shown in Fig. 3(c). Notice that this electron concentration of 3.7 × 10^18^ cm^−3^ is closed to that used in the Si JLTs^1, 18, 21^. Here we define the variation of channel resistance per gate voltage as follows:

1 $${\sigma }_{R}=\frac{1}{{\rm{\Delta }}{V}_{g}}{\rm{\Delta }}(\frac{{I}_{d}}{{V}_{d}})$$

**Figure 4:**
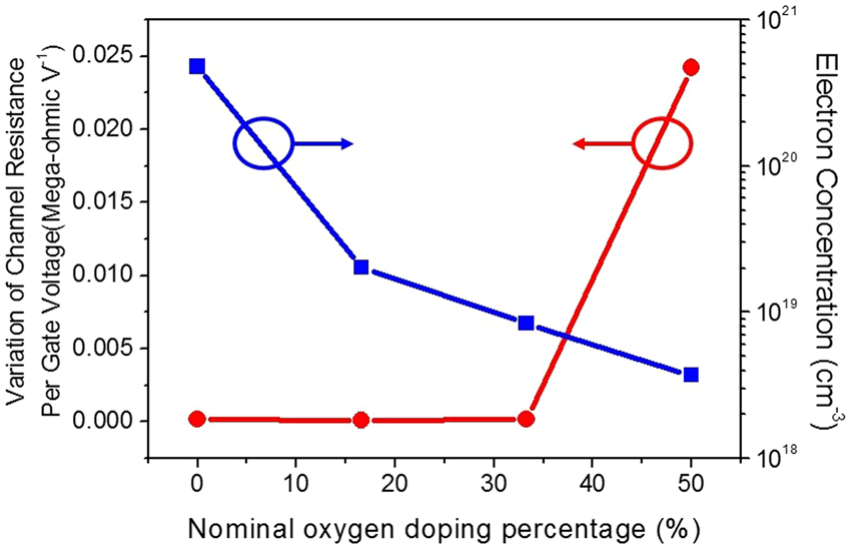
The variation of channel resistance per gate voltage *σ*_*R*_ and electron concentration of the 4.8 nm TiN-based ultrathin-body channel as a function of the nominal oxygen doping percentage.

If the channel can be controlled by the gate electric field, the variation of channel resistance will be nonzero. Figure 4 shows the *σ*_*R*_ and the electron concentration as a function of the *DP*_*O*_ from 0% to 50% in the 4.8 nm TiN-based ultrathin-body channels. The *σ*_*R*_ is zero with the *DP*_*O*_ from 0% to 33.3%, indicating that the external electric field can not extend into the TiN-based ultrathin-body channels to modulate the channel resistance because of the strong screening effect due to the high electron concentration. The increase of *DP*_*O*_ to 50% gives rise to nonzero *σ*_*R*_, clearly revealing the onset of field effect in the oxygen-doped TiN ultrathin-body channel as a result of the suppressed screening effect due to the low electron concentration. Figure 3(d) shows the *I*_*d*_ *-V*_*d*_ curves at various bottom gate voltages of the metallic channel transistor with the TiN-based channel, in which the channel thickness is 8 nm and the *DP*_*O*_ is 50%. As compared with Fig. 3(c), the modulation of *I*_*d*_ by the bottom gate voltage disappears in Fig. 3(d) with the increase of the channel thickness from 4.8 nm to 8 nm, which can be ascribed to the increase of the electron concentration from 3.7 × 10^18^ cm^−3^ to 3.3 × 10^19^ cm^−3^ (Fig. 2(c)). The oxygen-doped TiN channel recovers to metallic nature with the increasing film thickness from 4.8 nm to 8 nm even though the *DP*_*O*_ is up to 50%. The results revealed in Figs 3 and 4 demonstrate that the gate control and field effect can be achieved at room temperature in the TiN-based channel as long as the electron concentration is sufficiently low and the channel thickness is thin enough, which might be deduced from the reduced screening effect and the onset of quantum confinement.

**Figure 5:**
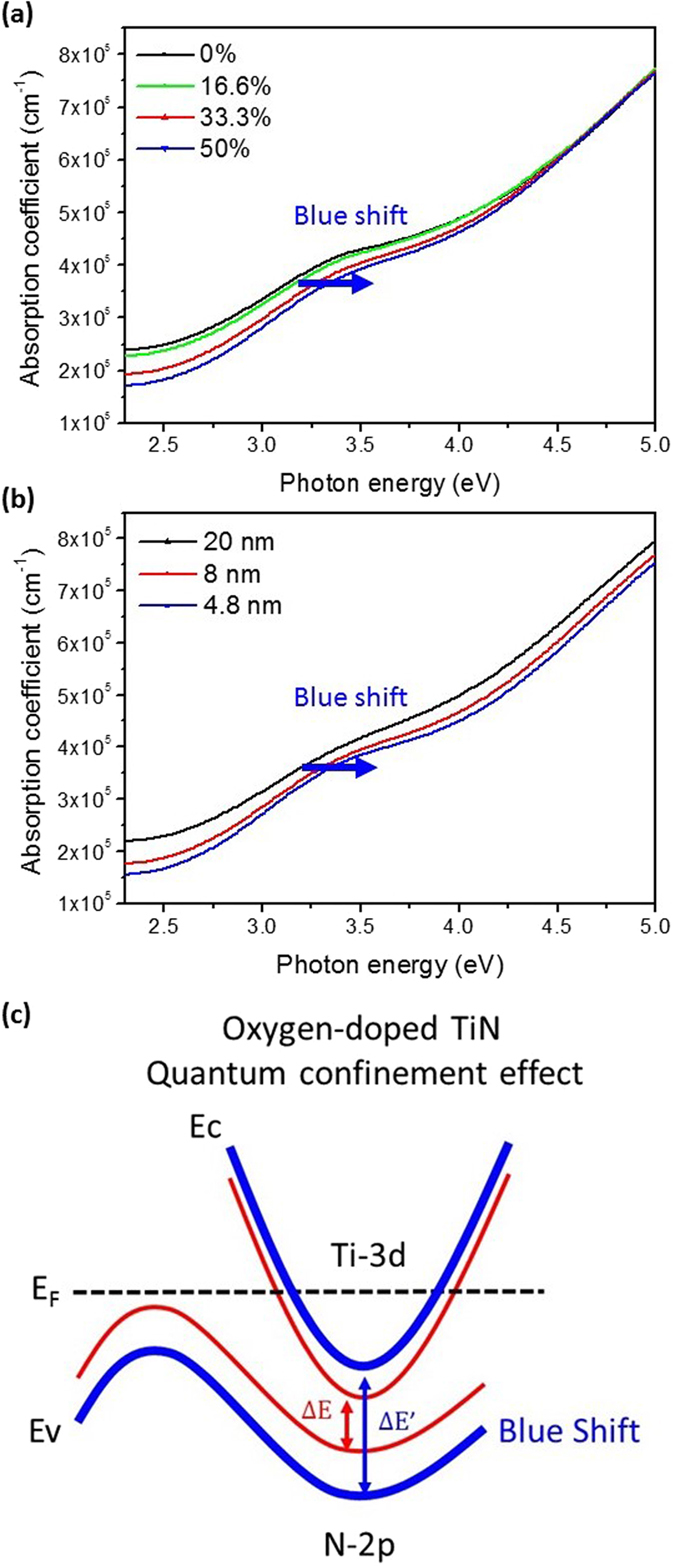
Absorption spectra of the oxygen-doped TiN layers. (**a**) The 20 nm TiN-based layers with the nominal oxygen doping percentage (*DP*_*O*_) from 0% to 50%. (**b**) The TiN-based layers of the *DP*_*O*_ = 50% with the thickness form 4.8 nm to 20 nm. (**c**) The schematic illustration of the increase of interband gap energy in TiN as a result of the oxygen incorporation and quantum confinement.

Figure 5 shows the absorption spectra of the TiN-based layers with different nominal oxygen doping percentages (*DP*_*O*_) and film thicknesses. The optical absorption at the photon energy greater than 2.3 eV is ascribed to the interband transitions between the 2*p* band of nitrogen and 3*d* band of titanium in TiN^40, 49, 50^. It is seen from Fig. 5(a) that the absorption spectra shifts toward the higher energy with the increasing *DP*_*O*_ from 0% to 50% in the 20 nm TiN-based layers, which suggests the rise of the interband gap energy with the increasing oxygen incorporation. In fact, the first-principles density-functional theory indicates that the 2*p-*3*d* interband gap energy of oxygen-doped TiN increases with the oxygen concentration^34^. The blue shift of the absorption spectra was also observed with the decreasing film thickness from 20 nm to 4.8 nm in the oxygen-doped TiN layers with the *DP*_*O*_ of 50%, as shown in Fig. 5(b), which may be understood as the quantum confinement effect^51–53^. Figure 5(c) shows the schematic diagram of the increase of interband gap energy in TiN due to the oxygen incorporation and the quantum confinement effect.

**Figure 6:**
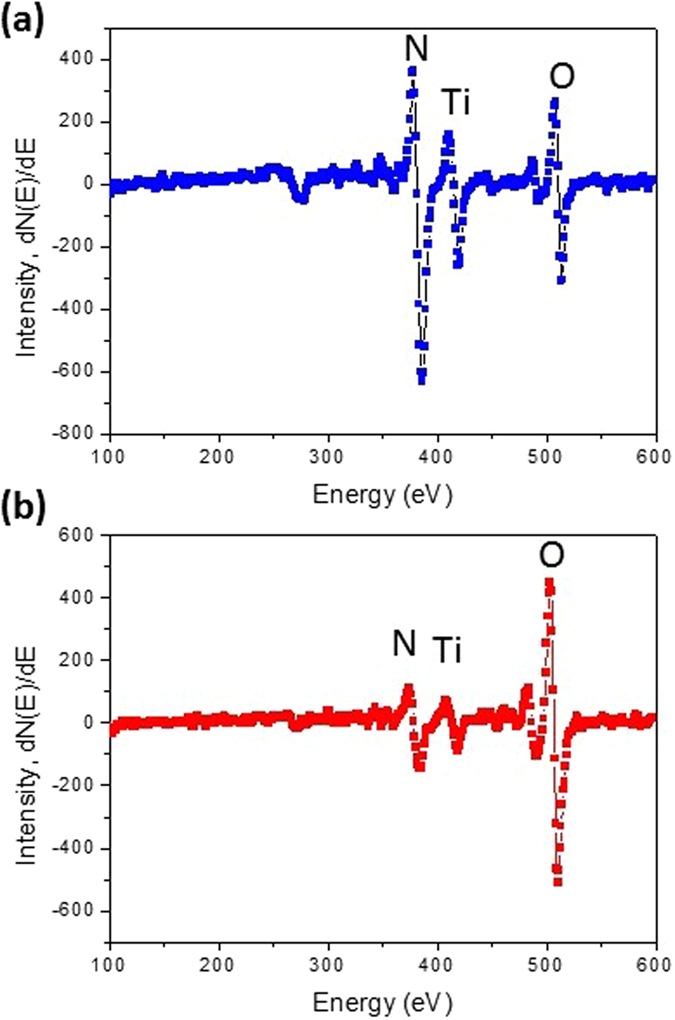
Auger survey spectra of the 20 nm TiN-based layers with the nominal oxygen doping percentage (*DP*_*O*_) of (**a**) 0% and (**b**) 50%.

The actual oxygen content in the oxygen-doped TiN layers were characterized by the AES measurement. Figure 6(a) and (b) show the nitrogen, oxygen, and titanium elements in the Auger survey spectra of the 20 nm TiN-based layers with the *DP*_*O*_ of 0% and 50%, which reveals the oxygen contents are approximately 5% and 35%, respectively. The ~5% oxygen content in the TiN-based layer without the *in-situ* atomic layer delta doping of oxygen (*DP*_*O*_ = 0%) may come from the residual oxygen contamination in the ALD chamber. The smaller oxygen content (35%) than the nominal oxygen doping percentage (*DP*_*O*_ = 50%) might be deduced from the incomplete chemical reaction in the process of the *in-situ* atomic layer delta doping in which the H_2_O was used as the oxidant^54–56^. Notice that the field effect and gate control occurred in the oxygen-doped TiN ultrathin-body channel with the oxygen content of 35% (*DP*_*O*_ = 50%) (Fig. 3(c)), which is close to the oxygen content (~40%) for the bandgap opening in the oxygen-doped TiN as predicted by the first-principles density-functional theory^34, 41^.

## Conclusion

The AL3D technique was proposed and utilized to precisely prepare the oxygen-doped TiN ultrathin-body channels in metallic channel transistors with accurate control of the channel thickness and electron concentration. With the sufficiently thin channel thickness (4.8 nm) and high oxygen content (35%), the channel exhibited the gate control and field effect at room temperature, which might be attributed to the onset of quantum confinement and the suppression of screening effect. Even though the conductivity modulation of an ultrathin metallic film is demonstrated at room temperature for the first time in this work, the TiN-based transistor does not turn off and essentially behaves as a resistor with non-saturation drain current. This result is ascribed to the fact that the conduction band and valence band are still overlapping in oxygen-doped TiN ultrathin-body channels, indicating that the novel metallic channel transistor prepared by the AL3D technique is still far from being accomplished and needs a lot of improvement to realize workable devices.
